# Observations in the Treatment of Sterility

**Published:** 1868-06-01

**Authors:** H. Webster Jones

**Affiliations:** 49 S. Ada Street


### OBSERVATIONS IN THE TREATMENT OF STERILITY.

BY H. WEBSTER JONES, M.D.

*Editor Chicago Journal:*

OPPORTUNITIES for the study of the causes and cure of sterility are so infrequent in these days, when men and women fashionably ignore the Creator's purpose in uniting them, that one is actually startled by a request for advice by which to further reproduction.

Such requests, quite recent, have led me to review the experience of a few years, and to present its results, as a disciple rather than teacher, to the readers of your journal.

In 1864 I was consulted by a lady, of perhaps thirty years, married and the mother of three children, the youngest of whom was then ten years of age. She was the subject of intense orbital neuralgia, of so peculiar a character as to demand the most careful investigation of the whole system, with reference to its ætiology. It was discovered that albuminuria was present to an alarming extent, and that the sufferings of the patient were enhanced by a retroversion of the uterus, supposed to have originated at the time of her last confinement. She had not conceived, to her knowledge, for ten years, though constantly in the society of her husband.

Among other attempts to relieve Mrs. H., I elevated the uterus upon the closed-bar lever pessary of Professor Hodge. Hitherto regular in menstruation, she became alarmed at the postponement of the very next expected period; was soon attended by all all her usual signs of pregnancy, with aggravation of her kidney disease, and much suffering from neuralgia, and finally aborted at three months, *involuntarily*.

I omit much of the interesting details of this case, remarking the chronic element in the albuminuria; the tolerably uniform recurrence of the neuralgia at monthly intervals, accompanied with well-marked *arcus senilis*, and lessening visual power; the reposition of the retroverted womb, followed immediately by pregnancy, the involuntary abortion, and last, the continuance of life and energy to the present date.

The patient always attributed her conception to the instrument used in elevating the womb, and objected to its reinsertion, averring her willingness to forego the relief and freedom of motion to which it for a time conduced, rather than to risk another pregnancy.

About the same time, I removed a pessary which had been worn by another patient, as one of a series, for the cure of retroversion, vaginal contraction, and vaginismus, following a protracted first labor, antedating about seven years. She assured me that connection with her husband had not taken place during the entire course of treatment, which occupied eight months. At the conclusion of this period, the uterus was nearly normal in its position, the vagina much elongated, and the vaginal hyperæsthesia subdued to a great extent. A single menstruation followed, when the patient discovered herself pregnant, and was duly delivered at term.

In the spring of 1865, Mrs. G., æt. twenty-three, having lost an only child of four years, consulted me with a view to the removal of her apparently sterile condition.

She had, unconsciously, a complete retroversion. It was treated mechanically, as in the first cases, and pregnancy followed the third menstruation after the instrument was introduced.

A year since, a friend consulted me upon his wife's health, and her prospect for child-bearing. She was thirty years of age, had never been rugged, and was never pregnant though married seven years. She had, a year previous, been quite ill, while away from home, I suppose with pelvic abscess, which finally found vent through the rectum. Her convalescence was tedious, accompanied by frequent hæmorrhages from the rectum, and her menses were dysmenhorrœic, exhausting and profuse.

She was "too weak to walk, or drive, or even stand for more than a few minutes at a time."

The womb was found prolapsed, resting persistently upon the rectum, which was swollen and tender, and the vagina was shortened by anterior displacement of the uterine axis.

Coition was said to be painful, from a sense of obstruction, and "every thing ran out," upon the conclusion of the marital embrace.

The womb was at once supported, and carried backward by a pessary (always Hodge's), astringent enemas ordered, and gentle exercise, to be followed by rest in recumbency enjoined.

At the completion of six months, the invalidism was overcome; the patient could walk, drive, stand "as well as ever," and the hæmorrhoids were rarely troublesome.

I removed the pessary, leaving the uterus well retired in the pelvis, and the vagina of normal dimensions. Pregnancy followed the second menstruation thereafter, and is now well advanced.

More recent investigations convince me of the truth of J. Marion Sims' position upon the inhibitory effect of uterine displacements in sterility. In three quite recent cases, the patients agree in relation to the difficulty of retaining the male discharges after coition. In each I find the uterus acting as a piston in a force pump, ejecting the vaginal contents immediately upon the withdrawal of the distending member.

Selecting the related cases because they are typical, little being done for them beside the mechanical aid mentioned, I purposely omit many others of doubtful origin, that I may suggest, if not prove, the relation of cause and effect between the anatomical fault and the co-existing sterility, inclining my readers as I hope, *pari passu*, to a candid investigation of the practical value of the curative means proposed.

As a summary, let me express a conviction of the usefulness of Hodge's closed-bar lever pessary, in the treatment of all such cases as are complicated by an obstructive anatomical arrangement of the pelvic organs, and more especially, whenever it is found that the piston-like descent of the uterus, and the spasmodic contraction of the distal vaginal walls forbid the retention of semen.

49 *S. Ada Street*, *May* 12*th*, 1868.

